# Lights on for Autism: Exploring Photobiomodulation as an Effective Therapeutic Option

**DOI:** 10.3390/neurolint14040071

**Published:** 2022-10-27

**Authors:** Catherine Hamilton, Ann Liebert, Vincent Pang, Pierre Magistretti, John Mitrofanis

**Affiliations:** 1 WellRed Pty Ltd., Launceston, TAS 7250, Australia; 2 Governance and Research Department, Sydney Adventist Hospital, Wahroonga, NSW 2076, Australia; 3 NICM Health Research Institute, University of Western Sydney, Penrith, NSW 2751, Australia; 4 Division of Biological and Environmental Sciences and Engineering, King Abdullah University of Science and Technology, Thuwal 2395, Saudi Arabia; 5 Fonds de Dotation Clinatec, Université Grenoble Alpes, 38000 Grenoble, France; 6 Institute of Ophthalmology, University College London, London WC1E 6BT, UK

**Keywords:** infrared, non-pharmacological, red, cell death, mitochondria

## Abstract

Autism is a neurodevelopmental condition that starts in childhood and continues into adulthood. The core characteristics include difficulties with social interaction and communication, together with restricted and repetitive behaviours. There are a number of key abnormalities of brain structure and function that trigger these behavioural patterns, including an imbalance of functional connectivity and synaptic transmission, neuronal death, gliosis and inflammation. In addition, autism has been linked to alterations in the gut microbiome. Unfortunately, as it stands, there are few treatment options available for patients. In this mini-review, we consider the effectiveness of a potential new treatment for autism, known as photobiomodulation, the therapeutic use of red to near infrared light on body tissues. This treatment has been shown in a range of pathological conditions-to improve the key changes that characterise autism, including the functional connectivity and survival patterns of neurones, the patterns of gliosis and inflammation and the composition of the microbiome. We highlight the idea that photobiomodulation may form an ideal treatment option for autism, one that is certainly worthy of further investigation.

## 1. Introduction

Autism spectrum disorder (referred henceforth to as “autism”) is characterised by two core symptoms; first, problems in social communication and interaction across contexts, and second, restricted, repetitive behaviours, interests, and activities [1]. It first becomes evident during early childhood, being about five times more prevalent in males than females, and follows into adulthood. Autism is clinically complex and the severity of symptoms varies widely between individuals given this diagnosis. It is associated frequently with various co-morbidities, including sensory and motor abnormalities, epilepsy, sleep disturbances, attention deficit and hyperactivity. Over recent years, the prevalence of autism has grown, with the current rate being approximately 1 in 160 [1,2,3,4,5,6,7,8,9].

Our aim in this mini-review is to consider the effectiveness of a potential new treatment for autism, known as photobiomodulation, the application of red to near infrared light (~λ = 600–1300 nm) on body tissues. Previous studies have shown that this treatment-in a range of animal models of disease improves the functional connectivity and survival patterns of neurones, the patterns of gliosis and inflammation and the composition of the microbiome, all of which characterise the key changes evident in autism. We highlight the idea that photobiomodulation can form an effective, safe, non-pharmacological and non-invasive treatment option for people with autism [10].

In the sections that follow, we will explore what is known currently of the neural mechanisms that underpin autism. We then discuss the current, and somewhat limited treatments available for the disorder. Finally, we consider the evidence that photobiomodulation improves many of the key cellular dysfunctions that characterise the disorder.

## 2. The Mechanisms

The factors responsible for the brain changes leading to an expression of autism are not entirely clear, but there is a strong genetic basis, with approximately 90% concordance for monozygotic twins. There is considerable heterogeneity in the genetics however, with no one single genetic mutation accounting for more than 1–2% of all cases; further, there are rich interactions between multiple genes and the environment, making things even more complex. Maternal nutrition, autoimmune disease and inflammation, and/or exposure to air pollutants (e.g., heavy metals) or various drugs (e.g., thalidomide or valproic acid) during preconception and pregnancy can aggravate a genetic problem or damage the brain, increasing the risk of autism [2,3,5,9].

**Figure 1:**
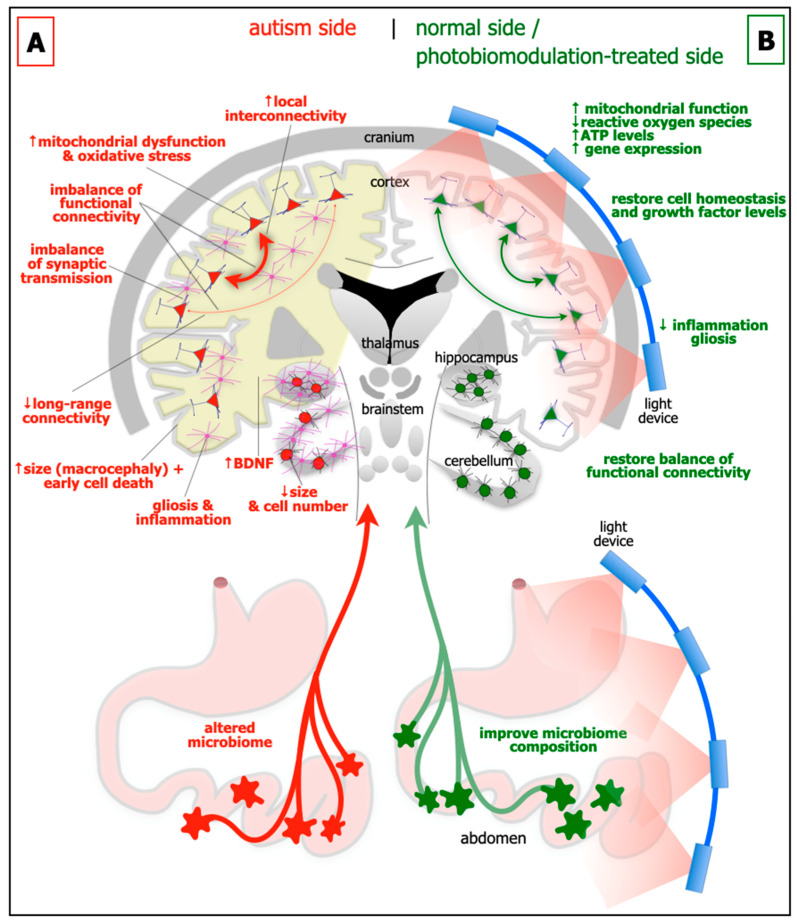
Schematic diagrams of the major abnormalities evident in autism (**A**; left side) as compared to normal, and after photobiomodulation treatment (**B**; right side). Autism is characterised by an altered microbiome in the gastrointestinal system (red star-like shapes), decrease in size of cerebellum and cerebellar cell number, increase in brain-derived neurotrophic factor (BDNF) levels in brain (yellow shade) and blood plasma, gliosis and inflammation in brain (pink cells), macrocephaly (increase in size of cortex), decrease in activity of long-range connectivity in cortex (thin red arrows), synaptic imbalance in brain, imbalance of functional connectivity, dysfunction and oxidative stress in brain (red cells) and increase in local interconnectivity in cortex (thick red arrows). We hypothesise that many if not all of these abnormalities will improve after photobiomodulation treatment to the head and to the abdomen (green cells and arrows). In particular, photobiomodulation will prompt; an increase in mitochondrial function, adenosine triphosphate (ATP) levels and gene expression, a reduction of oxidative stress, inflammation and gliosis, a restoration of cell homeostasis and growth factor levels, together with a restoration of a balanced functional activity across the brain.

Some of the major abnormalities in brain structure and function associated with autism are outlined below (Figure 1A).

**Brain size and cytoarchitecture abnormalities:** In approximately 20% of children with autism, there are general increases in the size of the cerebral cortex (ie brain overgrowth or macrocephaly), in particular the frontal, parietal and temporal areas. These early increases in size during childhood, appear to be followed by a premature decrease, presumably due to cell death (see below), from adolescence to late middle age [11,12]. But not all brain regions show the same patterns; the cerebellum for example, is generally smaller in children with autism (Figure 1A) [13,14]. Together with these abnormalities of size, there are distinct deficits in cytoarchitecture across different regions of the brain in autism. In the prefrontal cortex, particularly layer II, there are more neurones and fewer astrocytes, a feature linked to a developmental failure of radial glial cells to help immature neurones migrating to their appropriate cortical layer [15]. In the cerebellum, there are fewer cerebellar Purkinje and granule cells (Figure 1A) [13,14], while in the amygdala and hippocampus, there are differences in both size and in overall cell numbers [16,17].

**Functional connectivity imbalance:** There is an imbalance of functional connectivity across the brain in autism. These involve prefrontal, anterior cingulate, inferior parietal, and superior temporal cortices; these areas are associated with language, personality, task-switching, self-control, planning, working memory, social interactions and cognition, and many of the executive brain functions [18,19]. It has been suggested that autism can be characterised by an increased local interconnectivity but decreased long-range connectivity (Figure 1A) [20].

**Synaptic imbalance:** The balance of excitatory and inhibitory synaptic transmission is disrupted in autism (Figure 1A). A range of synaptic molecules and proteins become dysfunctional, such as those involved with cell adhesion [3]. There are decreased levels of glutamine and abnormal levels of glutamate evident in blood plasma [21], as well as many diverse glutamate receptors across the cortex [3]. There are reduced levels of glutamic acid decarboxylase, the rate-limiting enzyme in γ-aminobutyric acid (GABA) production, together with fewer GABA receptors [22]. A dysfunctional serotonergic system also contributes to the excitatory and inhibitory imbalance [3]; there are increased levels of serotonin in blood plasma and various genes encoding the serotonin neurotransmission are defective [23].

**Gliosis and inflammation:** There are clear signs of gliosis and inflammation in autism (Figure 1A). In both animal models and in people with autism, astrocytes and microglia-particularly in the hippocampus and the cerebellum-become reactive and release pro-inflammatory cytokines that exacerbate the inflammatory condition [9,24].

**Mitochondrial dysfunction and oxidative stress:** In autism, there is considerable mitochondrial dysfunction and oxidative stress, particularly in the cortex, hippocampus and cerebellum (Figure 1A). This results in increased levels of reactive oxygen species, an elevation of lipid peroxidation, abnormal calcium homeostasis and neurotransmitter imbalance, leading to dysfunctional neuronal activity and subsequent neuronal death [9,25].

**Growth factors:** A fascinating feature of autism is that there are elevated levels of growth factors in the brain, for example brain-derived neurotrophic factor (BDNF) in both the cortex and hippocampus (Figure 1A) [7], and in blood sera [26]. BDNF is a key molecule in maintaining cell homeostasis and function, and is associated with neuronal plasticity and growth. It has been suggested that elevated levels of BDNF generates synaptic dysfunction and is toxic to cells, leading to difficulties with executive function and behaviour [9,27,28]. Another view would be that the increase in BDNF in autism is a compensatory effect, in an attempt to repair the mitochondrial damage and cellular dysfunction, perhaps related to the increase in cell death during adolescence and middle age in autism (see above).

**Microbiome:** In addition to the changes evident in the brain, autism has also been linked to alterations in the gastrointestinal microbiome (Figure 1A) [8,9,29]. The microbiome is made up of microorganisms (i.e., bacteria, fungi, viruses, archaea, bacteriophages and protozoa) that reside, either transiently or permanently, within the gastrointestinal system. It has been described as the body’s additional or virtual organ, the key interface between food and the body. The microbiome has a number of critical functions, including: the digestion of food; increasing energy yields; contributing to nutrition; regulating sugar use and production and fat storage; together with influencing the integrity of the gut wall lining itself. Through its close relationship with the immune system and the large number of nerves that control the gut, the microbiome can have an enormous influence on many areas of health and well-being. Quite remarkably, during early development, the microbiome has been shown to influence brain networks and connectivity, particularly those related social interaction and behaviour; the key link in this relationship, namely the gut-brain axis, is through the far-reaching vagus nerve. In germ-free mice, those devoid of all microorganisms, there are alterations in protein and gene expression patterns across the brain, particularly within the hippocampus and amygdala. These changes are coupled with displays of abnormal social behaviours. If the microbiome composition is restored post-weaning in the mice, there is, rather strikingly, a reversal of these abnormalities. There are also indications that people with autism have altered microbiomes. For example, children with autism have been reported to have abnormal microbiome composition compared to controls; further, that autistic children often have gastrointestinal problems, with the severity related closely to the degree of behavioural disorder. The analysis of faecal matter from autistic children revealed a low relative abundance in a plethora of bacterial genera, including *Barnesiella*, *Parabacteroids*, *Alistipes putredinis*, *B. caccae*, *Bacteroides intestinihominis*, and the mucolytic bacterium *Akkermansia muciniphila* and *Bifidobacterium* spp. The consequential reduction in associated bacterial-derived genes encoding for key enzymes involved in the synthesis of GABA, melatonin and butyric acid, as well as possible alterations in gut mucosal barrier with pathological implications deriving from changes in gastrointestinal permeability, are all promising targets for novel treatment strategies [8,9,29].

## 3. Current Treatments

Autism is an extremely heterogeneous condition and management options depend on age, symptoms, behaviours, the individual’s own perception of their neurodiversity, and the nature and intensity of co-morbidities present. In early childhood, interventions that enhance parent-child interactions have been found to be helpful, such as strategies outlined in the Early Start Denver Model, and specific approaches designed to improve language function and challenging behaviours. At older ages, where professional support is available, the interventions are based on functional analysis of observed behaviour and evolving family situations, with a focus on intrinsic strengths and the development of more effective strategies to improve quality of life. These treatment strategies appear to only provide short-term improvements and current studies lack convincing evidence for their effectiveness in the long-term [30,31,32,33]. More recently, the role between the symptoms of autism and microbiome health has been targeted following the observation of distinct faecal and plasma metabolite profiles in children with autism. Early open-label studies have reported that faecal microbiota transplant in children with autism resulted in a shift in bacterial community in favour of the donor, indicating the possible promotion of donor microbe colonisation. Further, the Microbiota Transfer Therapy approach, consisting of a combination of antibiotics, bowel cleanse, stomach-acid suppressant, and faecal microbiota transplant, resulted in major improvements in gastrointestinal and autism-related symptoms, together with the overall composition of the gut microbiota. These observations were found to be maintained for two years following initial treatment, including an increase in bacterial diversity. Although promising, the availability of faecal transplantation and similar treatment modalities as a therapeutic option is currently extremely limited [8,9,29].

As for medications, there is only one currently drug approved by the United States Food and Drug Administration to treat the irritability symptoms and, to some extent, repetitive behaviours (i.e., risperidone), with some evidence supporting the benefits of aripiprazole. However, there is also evidence showing major adverse effects of these medications. There is no pharmaceutical intervention available to improve communication and social behaviours. Other drugs, for example selective serotonin re-uptake inhibitors, can be prescribed to help manage accompanying symptoms, such as anxiety and depression [4,5], but pharmaceutical treatments of co-morbidities tend to be used with caution because of autism’s clinical complexity [30,31,32,33,34,35].

Hence, there remains a real need for the development of a broad range treatment option that can be effective in a large number of people with autism. The treatment should ideally be non-invasive and non-pharmacological, as well as being easy to use with few or no side-effects.

## 4. Photobiomodulation: The Light

In this context, there is a potential new treatment option that has raised considerable interest across the community. This treatment has been shown, in a range of animal models of disease, as well as in humans, to influence the functional activity of neurones, creating a balanced pattern of neural connectivity to improve the survival of neurones after stress or damage (i.e., neuroprotective), and to reduce gliosis and inflammation [10,36,37]. Further, it has been shown to alter and improve microbiome diversity in both health and disease [38,39,40]. It has an impeccable safety record, with little or no evidence of side effects or toxicity on body cells, it is non-invasive and the devices are easy to use with high compliance. Taken all together, this treatment appears to “tick all the boxes” as an ideal treatment option for autism, one that is certainly worthy of further investigation. This treatment is known as photobiomodulation [10,36].

Photobiomodulation describes the non-invasive exposure of light, typically within the red to near-infrared (~λ = 600–1300 nm) spectrum, to elicit physiological effects across several tissue systems [10]. The effects of photobiomodulation exposure in this context, as an example of biostimulation, was first documented in 1967 by Endre Mester following an investigation using a 694 nm low powered laser, which resulted in accelerated hair growth in a mouse model [10]. Since then, photobiomodulation as a treatment modality has been well documented as a therapeutic intervention to include both coherent-light (lasers) or non-coherent light (light-emitting diodes, LEDs) [10].

Many studies from the last 70 years or so have reported that when neurones are under distress, photobiomodulation, after being absorbed by photoacceptors found mainly among the mitochondria, for example cytochrome oxidase c and/or interfacial nanowater, works to stimulate the production of ATP (adenosine triphosphate) energy that drives many intrinsic neuronal functions (Figure 1B). In addition, photobiomodulation also induces more long-term cellular changes, by activating the expression of various functional and protective genes. In essence, photobiomodulation makes the neurones “healthier”, by restoring their function and making them more resistant to distress. Photobiomodulation not only has a direct effect on neurones, but it also has an impact on reducing gliosis and/or inflammation (Figure 1B). Through these mechanisms, photobiomodulation has been reported to be disease-modifying or neuroprotective in a range of animal models of disease or trauma, from traumatic brain injury to stroke and from multiple sclerosis to Alzheimer’s and Parkinson’s disease [10,36,37,41].

When neurones are healthy and functioning normally, and there is no need to activate defence mechanisms, for example, the production of more energy and/or the expression of protective genes, photobiomodulation can still have an effect. In otherwise healthy neurones, there are many examples of photobiomodulation inducing either an increase [42,43,44,45,46,47] or a decrease in functional activity [42,48,49,50,51]. In the cortex, it has been suggested that photobiomodulation activates mechanisms that help focus attention or to help restore the overall balance of function and connectivity across any given system, particularly if it is dysfunctional (Figure 1) [52,53]. For example, in patients suffering from either traumatic brain injury or Alzheimer’s disease, both of which have abnormal patterns of functional connectivity between cortical areas, transcranial photobiomodulation helps correct these imbalances, restoring the connectivity between regions to “normal” levels [52,54].

In addition, there are some early observations that photobiomodulation, when applied to the abdomen, improves the function of the microbiome in normal healthy mice, as well as those treated with a toxin to induce Parkinson’s disease [38,40]. Further, when photobiomodulation is applied across the abdomen in both Alzheimer and Parkinson-induced disease mice, the death of brain cells associated with these conditions is very much reduced, indicating that an improved microbiome after photobiomodulation treatment can have a considerable impact on brain function and disease [38,40].

## 5. Effect of Photobiomodulation in Autism

Primary research investigating the safety and efficacy of photobiomodulation in autism have shown promising results.

There are several clinical reports using transcranial photobiomodulation in people with autism. Transcranial photobiomodulation treatment over an eight-week period has been reported to improve a range of behavioural measures, including social awareness, communication and motivation, and a reduction in restricted and repetitive behaviours [55]. In addition, transcranial photobiomodulation treatment for children with autism over a four week period reduced irritability and other symptoms [56]. These positive outcomes, quite remarkably, appear to be maintained for up to 12 months thereafter [57,58]. A placebo-controlled clinical trial using verum laser acupuncture indicates improvements in speech and social interactions in people with autism [59]. Further, the use of laser acupuncture in a child diagnosed with autism has been reported to generate a pattern of electroencephalography brain activity similar to that evident in normal children [60].

To the best of our knowledge, there are no animal studies exploring the effect of transcranial photobiomodulation in autism. Hence, we have no understanding of the functional and cellular effects that this treatment imparts on the autistic brain. There are however, two laser acupuncture studies, albeit using 405 nm light, a wavelength outside of the photobiomodulation range (~λ = 600–1300 nm), in a valproic acid animal model (see below). These studies report improvements in autistic-like behaviours and decreased measures of oxidative status in the cortex, hippocampus, striatum and cerebellum; within the cerebellum, there were also indications of increased GABAergic activity and Purkinje cell density [61,62].

## 6. A Working Hypothesis

Our working hypothesis (see Figure 1) is that photobiomodulation can be an effective therapeutic option in autism by:

(1) improving the behaviour and abnormal neural circuitry in the brain; we suggest that photobiomodulation will induce a more balanced pattern of functional connectivity between different regions of the brain;
(2) reducing cell death, mitochondrial dysfunction and oxidative stress, gliosis and inflammation in the brain; we propose that photobiomodulation will restore normal cell homeostasis;
(3) altering the composition of the microbiome and thence brain neural circuitry and thus behaviour; we suggest that microbial activity will be restored towards “normal” levels and that this will lead to an improvement in brain function.

Testing our hypothesis would require an animal model of autism. Although autism is a specific human disorder, there are a number of animal models that have been developed. None is considered to be a perfect representation of the human condition, as is indeed the case with all animal models for all human disorders and diseases, but they are nevertheless effective in generating some of the major features, namely many of the abnormal behaviours, neural circuitries and pathologies.

There are both genetic and chemically-induced (e.g., drug) types of models and these have proved invaluable for a better understanding of the mechanisms underpinning the disorder. The genetic models have focussed on mutations in cell surface protein genes-similar to those found in people with autism, while the chemically-induced models rely on exposing developing animals to certain chemicals. Perhaps the best-known model is the valproic acid-induced rodent model. Valproic acid is a broad-spectrum, anti-epileptic drug, but it is also a potent teratogen; when injected into pregnant or postnatal animals, it can induce a range of behavioural changes and pathologies similar to those evident in people with autism. These include: decreases in social behaviours and increases in repetitive behaviours; altered microbiome composition; excitatory-inhibitory synaptic imbalance; abnormal receptor expression and serotonin levels; abnormal growth of the cortex and cell migration patterns; gliosis and increased levels of pro-inflammatory cytokines; suppression of neurogenesis; and a reduction in cerebellar Purkinje cell number [2,3,4,5,9,63].

Hence, there are several well-established animal models for autism in which the effectiveness of photobiomodulation can be tested easily. Photobiomodulation could be applied-across the head and/or abdomen, both before (as a pre-treatment) and after (as a post-treatment) the development of the first behavioural signs and brain pathology. The pre-treatment could be a prophylactic, preventive approach, limiting the development and degree of brain dysfunction and symptoms; the post-treatment would be more in line with clinical reality, where people are treated after a diagnosis of autism and could be a reparative, restorative approach. A range of behaviours, together with an array of functional and molecular markers of neurones and glia in the brain-measuring cell death, mitochondrial function, oxidative stress, gliosis, inflammation and stress-could be tested. In addition, there could be a detailed measure of the changes in the microbiome composition. Using all these experimental approaches, a complete profile of the effects of photobiomodulation on different aspects of autism could be assembled. The collection of data, using these approaches, would confirm or refute our hypothesis.

With regard to use in humans, we suggest that, as a starting point, individuals with autism could use, on a daily basis, a transcranial photobiomodulation helmet; the daily use of photobiomodulation may improve the abnormal connectivity across the cortex, together with reducing the pathology and inflammation. Several types of helmets have been used successfully in, for example, patients with either Alzheimer’s [64] or Parkinson’s disease [65,66]; the parameters for these helmets include 670 nm and 810 nm wavelengths, set at a frequency of either 10 Hz or 40 Hz.

We should add that there would be no issue with the light from the photobiomodulation helmet device reaching through to the brain, at least to the superficial layers, including the cerebral cortex. Many previous studies have reported that photobiomodulation can penetrate from 30–50 mm of body tissues and most areas of the cortex are well within that range (~10–15 mm) [10,36]. Further, as an indication that the light from the photobiomodulation device can reach the brain, many studies have shown that light applied transcranially can change considerably the activity of neurones in the cerebral cortex (see above).

## 7. Conclusions

For people diagnosed with autism, there are few effective, broad range treatments available to treat the abnormal brain circuitry and microbiome environment, let alone the constellation and complexity of their symptoms. Recently, photobiomodulation has been shown to improve, for example in many animal models of Alzheimer’s to Parkinson’s disease, some of the key alterations of brain function and microbiome composition that are also found in autism. In addition, photobiomodulation is very safe, with little or no evidence of side effects or toxicity on body cells, it is non-invasive and the devices are easy to use with high compliance. Photobiomodulation appears to be an ideal treatment option for autism. Pre-clinical studies could be designed on animal models, establishing proof-of-concept, leading to a translation into use on people with autism and a large-scale clinical trial.

## Data Availability

Not applicable.
